# Efficacy of alternative or adjunctive measures to conventional treatment of peri-implant mucositis and peri-implantitis: a systematic review and meta-analysis

**DOI:** 10.1186/s40729-015-0023-1

**Published:** 2015-08-13

**Authors:** Frank Schwarz, Andrea Schmucker, Jürgen Becker

**Affiliations:** Department of Oral Surgery, Westdeutsche Kieferklinik, Universitätsklinikum Düsseldorf, D-40225 Düsseldorf, Germany

## Abstract

In patients with peri-implant mucositis and peri-implantitis, what is the efficacy of nonsurgical (i.e. referring to peri-implant mucositis and peri-implantitis) and surgical (i.e. referring to peri-implantitis) treatments with alternative or adjunctive measures on changing signs of inflammation compared with conventional nonsurgical (i.e. mechanical/ultrasonic debridement) and surgical (i.e. open flap debridement) treatments alone? After electronic database and hand search, a total of 40 publications (reporting on 32 studies) were finally considered for the qualitative and quantitative assessment. The weighted mean changes (WM)/ and WM differences (WMD) were estimated for bleeding on probing scores (BOP) and probing pocket depths (PD) (random effect model). Peri-implant mucositis: WMD in BOP and PD reductions amounted to −8.16 % [SE = 4.61] and −0.15 mm [SE = 0.13], not favouring adjunctive antiseptics/antibiotics (local and systemic) over control measures (*p* > 0.05). Peri-implantitis (nonsurgical): WMD in BOP scores amounted to −23.12 % [SE = 4.81] and −16.53 % [SE = 4.41], favouring alternative measures (glycine powder air polishing, Er:YAG laser) for plaque removal and adjunctive local antibiotics over control measures (*p* < 0.001), respectively. Peri-implantitis (surgical): WMD in BOP and PD reductions did not favour alternative over control measures for surface decontamination. WM reductions following open flap surgery (±resective therapy) and adjunctive augmentative therapy amounted to 34.81 and 50.73 % for BOP and 1.75 and 2.20 mm for PD, respectively. While mechanical debridement alone was found to be effective for the management of peri-implant mucositis, alternative/adjunctive measures may improve the efficacy over/of conventional nonsurgical treatments at peri-implantitis sites. Adjunctive resective and/or augmentative measures are promising; however, their beneficial effect on the clinical outcome of surgical treatments needs to be further investigated.

## Review

### Background

Peri-implant mucositis describes an inflammatory lesion that resides in the soft tissues compartment, while at peri-implantitis sites, this lesion has extended and also affects the implant supporting bone [1]. The 11th European Workshop on Periodontology has pointed to an “estimated weighted mean prevalence of peri-implant mucositis and peri-implantitis of 43 and 22 %, respectively” [2].

The main etiology of peri-implant mucositis refers to plaque accumulation [3, 4], and the conversion from mucositis to peri-implantitis was, particularly in the absence of a supportive maintenance care [5], positively correlated with the function time [2]. However, the presence of some independent systemic/patient-related (i.e. smoking) and local (i.e. residual cement, dimension of the keratinized tissue, surface roughness) risk indicators may increase the probability of the disease occurring [3].

According to the cause-related concept of therapy, professionally administered plaque removal is a key strategy for the prevention and management of peri-implant diseases [6]. In previous years, several alternative or adjunctive measures (e.g. local antibiotics, air polishing, laser application) have been proposed to improve the efficacy of nonsurgical treatment approaches [7–9]. At peri-implantitis sites, surgical protocols may involve different decontamination protocols, that may also be combined with resective (e.g. pocket elimination, bone re-contouring, implantoplasty) and/or augmentative approaches (e.g. bone substitutes or autografts with or without a supporting barrier membrane) [7, 10]. Accordingly, there is a need to identify the most effective interventions for the treatment of peri-implant diseases.

The aim of this systematic review was therefore to address the following focused question: in patients with peri-implant mucositis and peri-implantitis, what is the efficacy of nonsurgical (i.e. referring to peri-implant mucositis and peri-implantitis) and surgical (i.e. referring to peri-implantitis) treatments with alternative or adjunctive measures on changing signs of inflammation compared with conventional nonsurgical and surgical treatments alone?

### Methods

This systematic review was structured and conducted according to the preferred reporting items of the PRISMA statement [11].

#### Focused question

The focused question serving for literature search was structured according to the PICO format [12]: “In patients with peri-implant mucositis and peri-implantitis, what is the efficacy of nonsurgical (i.e. referring to peri-implant mucositis and peri-implantitis) and surgical (i.e. referring to peri-implantitis) treatments with alternative or adjunctive measures on changing signs of inflammation compared with conventional nonsurgical and surgical treatments alone?”.

Population: patients with peri-implant mucositis and peri-implantitis based on case definitions used in respective publications

Intervention: alternative or adjunctive measures to nonsurgical and surgical treatments

Comparison: conventional measures for nonsurgical and surgical treatments

Outcome: changes in peri-implant mucosal inflammation

#### Search strategy

The PubMed database of the U.S. National Library of Medicine and the Web of Knowledge of Thomson Reuters were used as electronic databases to perform a systematic search for relevant articles published in the dental literature between 1992 up to April 30, 2015. A commercially available software program (Endnote X7, Thomson, London, UK) was used for electronic title management. Screening was performed independently by two authors (F.S. and A.S.). Disagreement regarding inclusion during the first and second stage of study selection was resolved by discussion.

The combination of key words (i.e. Medical Subject Headings MeSH) and free text terms included:

“treatment” OR “nonsurgical treatment” OR “non-surgical treatment” OR “surgical treatment” OR “regenerative treatment” OR “augmentative treatment” OR “resective treatment” OR “reconstructive treatment” OR “therapy” OR “nonsurgical therapy” OR “non-surgical therapy” OR “surgical therapy” OR “regenerative therapy” OR “augmentative therapy” OR “resective therapy” OR “reconstructive therapy” OR “antiseptic treatment” OR “antibiotic treatment” OR “adjunctive treatment” OR “antiseptic therapy” OR “antibiotic therapy” OR “adjunctive therapy”

AND

“peri-implant disease” OR “periimplant disease” OR “peri-implant infection” OR “periimplant infection” OR “mucositis” (MeSH) OR “peri-implant mucositis” OR “periimplant mucositis” OR “Periimplantitis” (MeSH) OR “peri-implantitis”.

Electronic search was complemented by a hand search of the following journals:

- *Clinical Implant Dentistry and Related Research; Clinical Oral Implants Research; International Journal of Oral and Maxillofacial Implants; Journal of Clinical Periodontology; Journal of Periodontology*. Finally, the references of all selected full-text articles and related reviews were scanned. If required, the corresponding authors were contacted and requested to provide missing data or information.

#### Study inclusion and exclusion criteria

During the first stage of study selection, the titles and abstracts were screened and evaluated according to the following inclusion criteria:

1. English language.
2. Prospective randomized controlled (RCT), or non-randomized controlled (CCT) studies (split-mouth or parallel group designs) in humans comparing alternative (i.e. for biofilm removal) or adjunctive measures (i.e. for biofilm removal, or adjunctive antiseptic/antibiotic therapy, or regenerative/resective approaches) to conventional nonsurgical (i.e. mechanical/ultrasonic debridement) or surgical (i.e. open flap debridement) treatments.
3. Data on the clinical changes in mucosal inflammation (i.e. bleeding scores) and probing pocket depths (PD) following nonsurgical (referring to peri-implant mucositis and peri-implantitis) or surgical (referring to peri-implantitis) treatments in respective groups.

At the second stage of selection, all full-text articles identified during the first stage were acquired. During this procedure, the pre-selected publications were evaluated according to the following exclusion criteria:

1. Inclusion of less than five patients
2. Inadequate case definition [13]
3. Lack of clinical data on the changes in mucosal inflammation and PD

#### Quality assessment of selected studies

A quality assessment of all selected full-text articles was performed according to the Cochrane collaborations tool for assessing risk of bias (low, high, unclear) including the following domains: random sequence generation, allocation concealment, blinding of participants and personnel, blinding of outcome assessment, and incomplete outcome data [14]. Quality assessment was performed in two different phases. In particular, during phase I, quality assessment was based on the published full-text article performed independently by both authors. In phase II, disagreements were resolved by discussion.

#### Data extraction and method of analysis

A data extraction template was generated and based on the study design, population, case definition, observation period, interventions, comparisons, and primary and secondary outcomes as well as the study quality. For data analysis, the bleeding index (BI)/bleeding on probing (BOP) and PD scores after respective healing periods were defined as primary outcomes. Secondary outcomes included changes in marginal bone levels, immunological/microbiological parameters and the resolution of peri-implant diseases, based on case definitions used in respective publications. RCTs and CCTs not implementing appropriate control measures but reporting on changes in primary outcomes were used for the estimation of the overall efficacy of treatments.

Heterogeneity between RCTs, meta-analysis (i.e. weighted mean changes/differences and 95 % confidence intervals, random effect model to account for potential methodological differences between studies), forest plots and publication bias (Egger’s regression to quantify the bias captured by funnel plots) were assessed using a commercially available software program (Comprehensive Meta-Analysis V2, Biostat, Englewood, NJ 07631 USA).

### Results

#### Study selection

A total of 368 potentially relevant titles and abstracts were found during the electronic and manual search. During the first stage of study selection, 319 publications were excluded based on title and abstract. For the second phase, the complete full-text articles of the remaining 49 publications were thoroughly evaluated. A total of 19 papers had to be excluded at this stage because they did not fulfil the inclusion criteria of the present systematic review (Table 1).

**Table 1:** Excluded clinical studies at the second stage of selection and the reason for exclusion

Publication	Reason for exclusion
Lavigne et al. [63]	Experimental sites without BOP at baseline
Ciancio et al. [64]	Homecare plaque control protocol
Felo et al. [65]	Homecare plaque control protocol
Bach et al. [66]	Lack of clinical data defined for the present systematic review
Dörtbudak et al. [67]	Lack of a conventional control treatment
Khoury and Buchmann [68]	Changes in mucosal inflammation not reported
Roos-Jansaker et al. [69]	Observational study
Duarte et al. [70]	Observational study reporting on the same study population as [71]
Maximo et al. [71]	Observational study
Ramberg et al. [72]	Homecare plaque control protocol
Corbella et al. [73]	Prevention of peri-implant diseases
Heitz-Mayfield et al. [74]	Homecare plaque control protocol
Costa et al. [5]	Observational study
De Angelis et al. [75]	Lack of subgroup analyses
Salvi et al. [76]	Observational study
Deppe et al. [59]	Observational study
De Siena et al. [77]	Homecare plaque control protocol
McKenna et al. [78]	Case definition not reported
Flichy-Fernandez et al. [79]	Cross-over study design

**Fig. 1:**
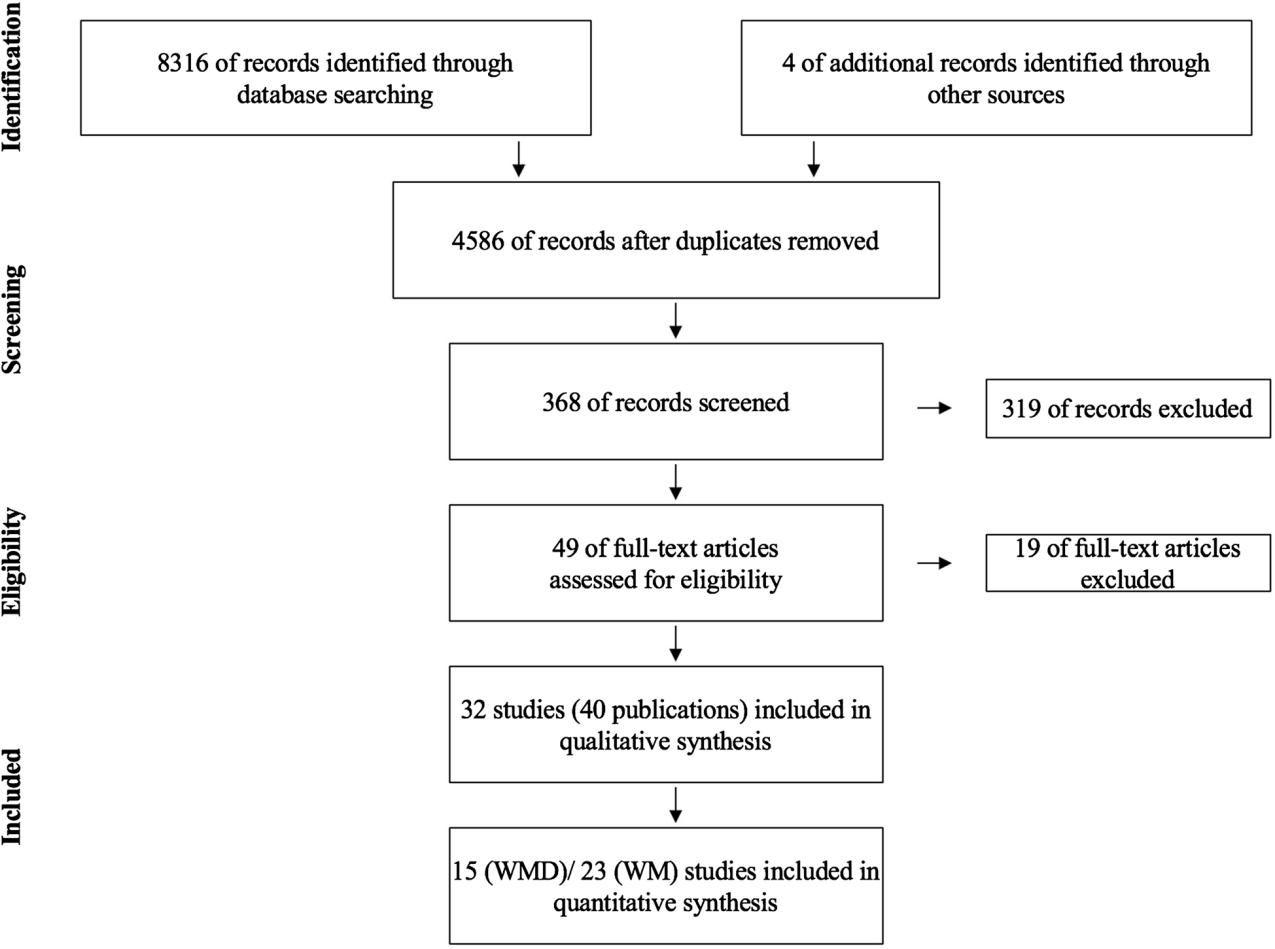
Flow diagram of literature search and inclusion

Finally, a total of 40 publications (reporting on 32 studies) were considered for the qualitative and quantitative assessment (Fig. 1).

#### Quality and risk of bias assessment of selected studies

The review author’s judgement about each risk of bias item for each included RCT is presented in Table 2. In particular, the percentages across all included studies for high, low and unclear risk of bias items were 34.1, 54.8 and 11.1 %, respectively (Table 2).

**Table 2:** Risk of bias summary for finally selected randomized studies

	Random sequence generation	Allocation concealment	Blinding of participants and personnel	Blinding of outcome assessment	Incomplete outcome data
Schenk et al. [16]	?	−	−	−	+
Strooker et al. [22]	?	−	−	−	+
Porras et al. [20]	?	−	−	+	−
Büchter et al. [26]	+	−	−	?	+
Romeo et al. [42, 57]	?	−	−	−	+
Karring et al. [28]	?	+	−	+	+
Schwarz et al. [25]	+	−	−	+	+
Renvert et al. [32]	+	+	−	+	+
Schwarz et al. [24]	+	−	−	+	+
Schwarz et al. [48, 51, 54]	+	−	−	+	+
Renvert et al. [29]	+	+	−	+	+
Renvert et al. [31]	+	?	−	+	+
Renvert et al. [30]	+	−	−	+	+
Sahm et al.; John et al. [27, 33]	+	−	−	+	+
Thone-Mühling et al. [17]	−	?	−	−	+
Hallström et al. [19]	+	?	−	−	+
Schwarz et al. [49, 50, 52]	+	−	−	+	+
Aghanzadeh et al. [43]	?	?	−	+	+
Machtei et al. [23]	+	+	?	+	+
Wohlfahrt et al. [55, 58]	+	+	−	+	+
deWaal et al. [38]	+	+	?	+	+
McKenna et al. [78]	+	−	+	+	−
Schär et al.; Bassetti et al. [34, 37]	+	−	−	+	
deWaal et al. [39]	+	+	?	+	+
Ji et al. [15]	+	?	−	+	+
Papadopoulos et al. [41]	+	−	−	+	+
Riben Grundström et al. [21]	+	+	−	+	+

+ low, − high, ? unclear

#### Subdivision of selected studies

All selected publications were subdivided according to differences in the treatment protocol:

- o Nonsurgical treatment of peri-implant mucositis—alternative or adjunctive measures for biofilm removal (2 RCTs and 1 CCT) (Table 3)

**Table 3:** Included studies—nonsurgical treatment of peri-implant mucositis: alternative or adjunctive measures for biofilm removal

Publication	Design	Population	Case definition	Period	Test	Control	Mean (SD) outcome
Ji et al. [15]	RCT, parallel	24 patients	PD ≥4 mm, BOP + no radiographic bone loss compared with baseline (i.e. immediately after prosthesis installation)	3 months	OHI + mechanical debridement (ultrasonic scaler with carbon fibre tips) + air abrasive device, glycine powder (sites with PD ≥4 mm)	OHI + mechanical debridement (ultrasonic scaler with carbon fibre tips)	Test
		33 implants					BI: 1.4 (0.57) (BL) to 1.1 (0.58) (3 months, subject level)
		Molar/premolar sites					BI: 1.7 (0.93) (BL) to 1.1 (0.98) (3 months, implant level)
		1 implant system					Sites without bleeding: 29.3 %
							PD: 3.6 (0.47) (BL) to 3.2 (0.48) mm (3 months, subject level)
							Control
							BI: 1.5 (0.65) (BL) to 1.0 (0.85) (3 months, subject level)
							BI: 1.7 (1.0) (BL) to 0.9 (1.1) (3 months, implant level)
							Sites without bleeding: 42 %
							PD: 3.5 (0.5) (BL) to 3.1 (0.38) mm (3 months, subject level)
De Siena et al. [18]	CCT, parallel	30 patients	BOP or spontaneous bleeding with local swelling	6 months	OHI + mechanical debridement Teflon curettes, polishing) + air abrasive device, glycine powder	OHI + mechanical debridement Teflon curettes, polishing)	Test
		No information on number and types of implants	PD ≤3.5 mm				PD: 3.0 (0.4) (BL) to 2.4 (0.5) mm (6 months, subject level)
			Bone loss ≤ 3.0 mm				13 patients did not present bleeding at 6 months
							Control
							PD: 2.9 (0.4) (BL) to 3.0 (0.6) mm (6 months, subject level)
							9 patients did not present bleeding at 6 months
							BI and PD scores sign. lower in the test group at 6 months
Riben Grundström et al. [21]	RCT, parallel	37 patients	PD ≥4 mm, BOP + with or without suppuration	12 months	OHI + air abrasive device, glycine powder	OHI + mechanical debridement (ultrasonic scaler with plastic coated tips)	Test
		One implant per subject used	Bone loss ≤2 mm from implant shoulder		Repeated treatment at 3 and 6 months	Repeated treatment at 3 and 6 months	BOP: 43.9 (7.3) (BL) to 12.1 (3.8) % (12 months, subject level)
		Test *N* = 19					Number of diseased sites (pocket depth ≥4 mm with bleeding/suppuration)
		Control *N* = 18					38 % (BL) to 8 % (12 months, subject level)
							Control
							BOP: 53.7 (7.9) (BL) to 18.6 (6.4) % (12 months, subject level)
							Number of diseased sites (pocket depth ≥4 mm with bleeding/suppuration)
							52 % (BL) to 17 % (12 months, subject level)
							No significant differences between groups for either reduction of
							BOP or of diseased sites

*BI* bleeding index, *BL* baseline, *BOP* bleeding on probing, *CCT* non-randomized controlled clinical study, *OHI* oral hygiene instructions, *PD* probing pocket depth, *RCT* randomized controlled clinical study
- o Nonsurgical treatment of peri-implant mucositis—adjunctive antispectic therapy (3 RCTs) (Table 4)

**Table 4:** Included studies—nonsurgical treatment of peri-implant mucositis: adjunctive antiseptic therapy

Publication	Design	Population	Case definition	Period	Test	Control	Mean (SD) outcome
Strooker et al. [22]	RCT Split-mouth design	16 patients each with 4 mandibular implants and bar retained over denture	Not reported	5 months	Supra-/subgingival scaling (carbon curettes) + polishing (rubber cup) + phosphoric acid gel (35 %) in sulcus for 1 min	Supra-/subgingival scaling (carbon curettes) + polishing (rubber cup) Once every month	Test (subject level)
					Once every month		BOP: 30.5 (27.5) (BL) to 9.7 (10.97) % (5 months)
							GI: 0.92 (0.75) (BL) to 0.34 (0.38) (5 months)
							PD: 2.97 (0.68) (BL) to 2.34 (0.54) mm (5 months)
							Control (subject level)
							BOP: 29.2 (29.44) (BL) to 14.3 (22.47) % (5 months)
							GI: 0.82 (0.8) (BL) to 0.57 (0.6) (5 months)
							PD: 2.83 (0.57) (BL) to 2.48 (0.49) mm (5 months)
							Sign. between group difference in mean GI values and colony-forming units at 5 months
Porras et al. [20]	RCT, parallel	16 patients	Supra- and subgingival plaque	3 months	OHI + mechanical cleansing (plastic scaler, rubber cups, polishing paste) + local irrigation CHX (0.12 %) and topical CHX gel application + 0.12 % CHX mouthrinse twice for 10 days	OHI + mechanical cleansing (plastic scaler, rubber cups, polishing paste)	mBI and BOP (%) scores: no sign. differences between groups at 1 and 3 months
		28 implants	PD ≤5 mm BOP + “incipient” radiographic lesion				PD values:
		3 implant types (plasma-sprayed Ti/cp Ti (HA-coated Ti)					Test: 3.27 (0.81) (BL) to 2.71 (0.70) mm (3 months)
							Control: 3.48 (0.61) (BL) to 2.55 (0.72) mm (3 months)
							Changes in mean PD between test and control groups at 3 months were statistically significant (0.56 vs. 0.93 mm)
							Microbiological improvements in both groups
Thone-Mühling et al. [17]	RCT, parallel	11 patients	BOP + and/or GI ≥1 absence of radiographic bone loss during the last 2 years	8 months	OHI + mechanical cleansing (plastic scaler and polyetheretherketone-coated ultrasonic instruments) + topical CHX gel application once + full mouth disinfection (deep scaling in one session + CHX disinfection of tongue and tonsils) + 0.2 % CHX mouthrinse 2×/day and tonsil spraying 1×/day for 14 days	OHI + mechanical cleansing (plastic scaler and polyetheretherketone-coated ultrasonic instruments) + full mouth scaling in one session	Test
		36 implants					BOP: 0.22 (0.11) (BL) to 0.16 (0.09) % (8 months)
		2 implant types					GI: 0.6 (0.24) (BL) to 0.44 (0.23) (8 months)
							PD: 3.4 (0.68) (BL) to 2.82 (0.59) mm (8 months)
							Control
							BOP: 0.17 (0.19) (BL) to 0.17 (0.11) % (8 months)
							GI: 0.62 (0.36) (BL) to 0.43 (0.37) (8 months)
							PD: 3.49 (0.78) (BL) to 2.84 (0.64) mm (8 months)
							Bacterial recolonization over time

*BL* baseline, *BOP* bleeding on probing, *GI* modified gingival index, *mBI* modified bleeding index, *OHI* oral hygiene instructions, *PD* probing pocket depth, *RCT* randomized controlled clinical study
- o Nonsurgical treatment of peri-implant mucositis—adjunctive antibiotic therapy (2 RCTs) (Table 5).

**Table 5:** Included studies—nonsurgical treatment of peri-implant mucositis: adjunctive antibiotic therapy

Publication	Design	Population	Case definition	Period	Test	Control	Mean (SD) outcome
Schenk et al. [16]	RCT Split-mouth design	8 patients	PD >4 mm BOP on at least 1 site per implant ± mucosal hyperplasia no radiographic bone loss	3 months	Supra-/subgingival scaling (steel curettes) + polishing (rubber cup) + locally delivered tetracycline HCl (25 %) fibre for 10 days +0.2 % CHX mouthrinse twice for 10 days	Supra-/subgingival scaling (steel curettes) + polishing (rubber cup) + +0.2 % CHX mouthrinse twice for 10 days	ΔBOP (3 months, subject level)
		24 implants					Test: −17 ± 25 %
		1 implant type (endossous part: titanium and zirconoxide/transmucosal part: titanium oxinitride)					Control: 15 ± 37 %
							PD/CAL values without significant changes in both groups
							No adverse events
							Partial/complete fibre loss at three sites
Hallström et al. [19]	RCT, parallel	45 patients	PD ≥4 mm BOP + and/or pus	6 months	OHI + mechanical cleansing (titanium curettes + rubber cups + polishing paste) + Azithromycin® 500 mg day 1 and 250 mg days 2–4	OHI + mechanical cleansing (titanium curettes + rubber cups + polishing paste)	Test
		3 implant systems	Radiographic bone loss ≤2 mm				BOP: 82.6 (24.4) (BL) to 27.3 (18.8) % (6 months, subject level)
							PD at worst site: 5.5 (0.8) (BL) to 4.1 (1.2) mm (6 months, subject level)
							Control
							BOP: 80.0 (25.0) (BL) to 47.5 (32.3) % (6 months, subject level)
							PD at worst site: 5.7 (0.8) (BL) to 4.9 (1.1) mm (6 months, subject level)
							Odds ratio of a positive treatment outcome (PD ≤ 4.0 mm and BOP ≤ 1) was 4.5:1 (test vs. control)
							Comparable reductions in bacterial counts

*BL* baseline, *BOP* bleeding on probing, *CAL* clinical attachment level, *OHI* oral hygiene instructions, *PD* probing pocket depth, *RCT* randomized controlled clinical study
- o Nonsurgical treatment of peri-implantitis—alternative measures for biofilm removal (6 RCTs) (Table 6)

**Table 6:** Included studies—nonsurgical treatment of peri-implantitis: alternative measures for biofilm removal

Publication	Design	Population	Case definition	Period	Test	Control	Mean (SD) outcome
Karring et al. [28]		11 patients	PD ≥5 mm, BOP + bone loss >1.5 mm and exposed threads	3 months	OHI + ultrasonic device with hydroxyapatite fluid polish	OHI + mechanical debridement (carbon fibre curettes)	Test
		22 implants machined and medium-rough surfaces					BOP: 63.6 (BL) to 36.4 % (3 months, subject level)
							PD: 5.8 (1.1) (BL) to 5.8 (1.2) mm (3 months, subject level)
							Radiographic bone level changes: 0.1 (0.5) mm (3 months, subject level)
							Control
							BOP: 72.7 (BL) to 81.8 % (3 months, subject level)
							PD: 6.2 (1.6) (BL) to 6.3 (2.2) mm (3 months, subject level)
							Radiographic bone level changes: −0.2 (1.2) mm (3 months, subject level)
							Absence of BOP at 7/11 (test) and 2/11 (control) sites
Schwarz et al. [25]	RCT, parallel	20 patients	PD ≥4 mm, BOP + and pus	6 months	OHI + Er:YAG laser device (cone-shaped glass fibre tip) at 12.7 J/cm^2^	OHI + mechanical debridement (plastic curettes), 0.2 % CHX pocket irrigation and 0.2 % CHX gel	Test
		32 implants rough and medium-rough surfaces	Radiographic bone loss				BOP: 83.2 (17.2) (BL) to 31.1 (10.1) % (6 months, subject level)
							PD: 5.4 (1.2) (BL) to 4.6 (1.1) mm (6 months, subject level)
							Control
							BOP: 81.3 (19.0) (BL) to 58.3 (16.9) % (6 months, subject level)
							PD: 5.5 (1.5) (BL) to 4.8 (1.4) mm (6 months, subject level)
							BOP scores at 6 months were significantly lower in the test group
Schwarz et al. [24]	RCT, parallel	20 patients	PD ≥4 mm, BOP + and pus	12 months	OHI + Er:YAG laser device (cone-shaped glass fibre tip) at 12.7 J/cm^2^	OHI + mechanical debridement (plastic curettes), 0.2 % CHX pocket irrigation and 0.2 % CHX gel	Test
		40 implants rough and medium-rough surfaces	Radiographic bone loss				Moderately deep sites
							BOP: 81.7 (6.7) (BL) to 35.0 (6.3) % (12 months, subject level)
							PD: 4.5 (1.4) (BL) to 4.0 (0.1) mm (12 months, subject level)
							Deep sites
							BOP: 79.9 (4.8) (BL) to 55.0 (6.5) % (12 months, subject level)
							PD: 5.9 (0.1) (BL) to 5.4 (0.1) mm (12 months, subject level)
							Control
							Moderately deep sites
							BOP: 81.6 (5.2) (BL) to 53.3 (7.3) % (12 months, subject level)
							PD: 4.4 (0.2) (BL) to 4.3 (0.1) mm (12 months, subject level)
							Deep sites
							BOP: 88.3 (3.5) (BL) to 66.6 (5.5) % (12 months, subject level)
							PD: 5.9 (0.3) (BL) to 5.5 (0.2) mm (12 months, subject level)
							No significant differences between groups at 12 months
Renvert et al. [31]	RCT, parallel	31 patients	PD ≥4 mm, BOP + and/or pus	6 months	OHI + ultrasonic device with hydroxyapatite fluid polish	OHI + mechanical debridement (titanium curettes)	Test
		31 implants machined and medium-rough surfaces	Bone loss <2.5 mm				BI: 1.7 (0.6) (BL) to 1.2 (0.7) (6 months, subject level)
							PD: 4.3 (0.6) (BL) to 3.9 (0.8) mm (6 months, subject level)
							Control
							BI: 1.7 (0.9) (BL) to 1.4 (1.0) (6 months, subject level)
							PD: 6.2 (1.6) (BL) to 6.3 (2.2) mm (6 months, subject level)
							No significant differences between groups
Renvert et al. [30]	RCT, parallel	42 patients	PD ≥5 mm, BOP + and/or pus	6 months	OHI + air abrasive device, glycine powder	OHI + Er:YAG laser device (cone-shaped glass fibre tip, 12.7 J/cm^2^)	Test
		90 implants machined and medium-rough surfaces	Bone loss >3 mm				PD changes: 0.9 (0.8) mm (6 months, implant level)
							Radiographic bone level change: −0.3(0.9) mm (6 months, subject level)
							Positive treatment outcome: 47 %
							Control
							PD changes: 0.8 (0.5) mm (6 months, implant level)
							Radiographic bone level change: −0.1(0.8) mm (6 months, subject level)
							Positive treatment outcome: 44 %
							No significant differences between groups
Sahm et al.; John et al. [27, 33]	RCT, parallel	32 patients (BL)	PD ≥4 mm, BOP + with suppuration	12 months	OHI + air abrasive device, glycine powder	OHI + mechanical debridement (carbon curettes + 0.1 % CHX)	Test
		25 patients (12 months)	Bone loss ≤33 %				BOP: 99.0 (4.1) (BL) to 57.8 (30.7) % (12 months, subject level)
		36 implants					PD: 3.7 (1.0) (BL) to 3.2 (1.1) mm (12 months, subject level)
		8 implant systems					Control
							BOP: 94.7 (13.7) (BL) to 78.1 (30.0) % (12 months, subject level)
							PD: 3.9 (1.1) (BL) to 3.5 (1.2) mm (12 months, subject level)
							BOP: significant difference between groups at 3, 6 and 12 months

*BI* bleeding index, *BL* baseline, *BOP* bleeding on probing, *CHX* chlorhexidine digluconate, *OHI* oral hygiene instructions, *PD* probing pocket depth, *RCT* randomized controlled clinical study
- o Nonsurgical treatment of peri-implantitis—adjunctive antiseptic therapy (1 RCT) (Table 7)

**Table 7:** Included studies—nonsurgical treatment of peri-implantitis: adjunctive antiseptic therapy

Publication	Design	Population	Case definition	Period	Test	Control	Mean (SD) outcome
Machtei et al. [23]	Multicentre RCT, parallel	60 patients	PD = 6–10 mm and BOP + radiographic bone loss	6 months	OHI + ultrasonic debridement + matrix containing 2.5-mg CHX chips (i.e. up to 4 per implant site)	OHI + ultrasonic debridement + biodegradable crosslinked gelatin matrix chip	Test
		77 implants			Repeated application at sites with PD ≥6 mm at 2, 4, 6, 8, 12 and 18 weeks	Repeated application at sites with PD ≥6 mm at 2, 4, 6, 8, 12 and 18 weeks	BOP: 100 (0.0) (BL) to 42.5 (50.0) % (6 months, subject level)
							PD: 7.6 (1.1) (0.0) to 5.47 (1.86) mm (6 months, subject level)
							Control
							BOP: 100 (0.0) (BL) to 54.5 (50.5) % (6 months, subject level)
							PD: 7.21 (1.08) (BL) to 5.48 (1.25) mm (6 months, subject level)
							BOP and PD reductions at 6 months not sign. different between groups

*BL* baseline, *BOP* bleeding on probing, *CHX* chlorhexidine digluconate, *OHI* oral hygiene instructions, *PD* probing pocket depth, *RCT* randomized controlled clinical study
- o Nonsurgical treatment of peri-implantitis—adjunctive antibiotic therapy (4 RCTs) (Table 8)

**Table 8:** Included studies—nonsurgical treatment of peri-implantitis: adjunctive antibiotic therapy

Publication	Design	Population	Case definition	Period	Test	Control	Mean (SD) outcome
Büchter et al. [26]	RCT, parallel	28 patients	PD >5 mm	18 weeks	OHI + mechanical debridement (plastic curettes) + 0.2 % CHX pocket irrigation + 8 % doxycycline hyclate	OHI + mechanical debridement (plastic curettes) + 0.2 % CHX pocket irrigation	Test
		48 implants medium-rough surfaces	Bone loss >50 %				BOP: 0.54 (0.07) (BL) to 0.27 (0.06) % (18 weeks, subject level)
							PD: 5.64 (0.32) (BL) to 4.49 (0.29) mm (18 weeks, subject level)
							Control
							BOP: 0.63 (0.06) (BL) to 0.50 (0.07) % (18 weeks, subject level)
							PD: 5.68 (0.28) (BL) to 5.4 (0.34) mm (18 weeks, subject level)
							BOP and PD reductions sign. higher in the test group
Renvert et al. [32]	RCT, parallel	32 patients	PD ≥4 mm, BOP + with suppuration	12 months	OHI + mechanical debridement (scalers + rubber cup + polishing) + 1 mg minoycycline microspheres	OHI + mechanical debridement (scalers + rubber cup + polishing) + 1.0 % chlorhexidine gel	Test
		1–5 (test)/1–6 (control) implants per patient machined surfaces	Bone loss ≤3 threads				BOP: 88 (12) (BL) to 71 (22) % (12 months, subject level)
			Presence of anaerobic bacteria				PD: 3.9 (0.7) (BL) to 3.6 (0.6) mm (12 months, subject level)
							Control
							BOP: 86 (14) (BL) to 78 (13) % (12 months, subject level)
							PD: 3.9 (0.3) (BL) to 3.9 (0.4) mm (12 months, subject level)
							PD reductions at 12 months sign. higher in the test group
							Comparable microbiological improvements in both groups
Renvert et al. [29]	RCT, parallel	32 patients	PD ≥4 mm, BOP + with suppuration	12 months	OHI + mechanical debridement + 1 mg minoycycline microspheres	OHI + mechanical debridement + 0.5 ml of 1.0 % CHXgel	Test
		95 implants machined surfaces	Bone loss ≤3 threads		Treatment was repeated at days 30 and 90	Treatment was repeated at days 30 and 90	BOP: 86.5 (20.1) (BL) to 48.1 (20.7) % (12 months, implant level)
			Presence of anaerobic bacteria				PD: 3.85 (1.04) (BL) to 3.55 (0.98) mm (12 months, implant level)
							Radiographic bone levels: 0.77 (0.85) (BL) to 0.7 (0.85) mm (12 months, implant level)
							Control
							BOP: 89.2 (17.2) (BL) to 63.5 (19.2) % (12 months, implant level)
							PD: 3.87 (1.16) (BL) to 3.72 (1.02) mm (12 months, implant level)
							Radiographic bone levels: 0.41 (0.7) (BL) to 0.46 (0.76) mm (12 months, implant level)
							BOP reductions at 12 months sign. higher in the test group
							Comparable microbiological improvements in both groups
Schär et al.; Bassetti et al. [34, 37]	RCT, parallel	40 patients	PD = 4–6 mm, BOP + bone loss = 0.5–2 mm	12 months	OHI + mechanical debridement (titanium curettes + glycin powder air polishing, pocket irrigation using 3 % hydrogen peroxide) + aPDT (660 nm, phenothiazine chloride dye)	OHI + mechanical debridement (titanium curettes + glycin powder air polishing, pocket irrigation using 3 % hydrogen peroxide) + minocycline microspheres	Test
		40 implants medium-rough surfaces					BOP change: 57 % (12 months, subject level)
							PD changes: 0.56 mm (12 months, subject level)
							Complete resolution of mucosal inflammation: 31.6 %
							Control
							BOP change: 65 % (12 months, subject level)
							PD changes: 0.11 mm (12 months, subject level)
							Complete resolution of mucosal inflammation: 35.0 %
							No significant differences in clinical, microbiological and immunological parameters between groups

*aPDT* antimicrobial photodynamic therapy, *BL* baseline, *BOP* bleeding on probing, *CHX* chlorhexidine digluconate, *OHI* oral hygiene instructions, *PD* probing pocket depth, *RCT* randomized controlled clinical study
- o Surgical treatment of peri-implantitis—alternative measures for surface decontamination (3 RCTs and 1 CCT) (Table 9)

**Table 9:** Included studies—surgical treatment of peri-implantitis: alternative measures for surface decontamination

Publication	Design	Population	Case definition	Period	Test	Control	Mean (SD) outcome
Deppe et al. [40]	CCT, parallel	32 patients	PD ≥5 mm, BOP + progressive vertical bone loss	5 years	OHI + open flap^a^ surgery + air polishing + carbon dioxide laser (cw mode, 2.5 W, 12 × 5 s) decontamination + soft tissue resection	OHI + open flap^b^ surgery + air polishing + soft tissue resection	Test
		73 implants machined, rough- and medium-rough surfaces					SBI: 0.6 (0.3) (BL) to 1.8 (1.1) (48 months, implant level)
							PD: 6.1 (1.6) (BL) to 3.4 (1.5) mm (48 months, implant level)
							Control
							SBI: 0.7 (0.8) (BL) to 1.1 (1.2) (48 months, implant level)
							PD: 5.1 (1.3) (BL) to 4.3 (1.2) mm (48 months, implant level)
							Comparable outcomes in both groups
De Waal et al. [38]	RCT, parallel	30 patients	PD ≥5 mm, BOP + and/or suppuration	12 months	OHI/mechanical debridement + resective therapy (apical re-positioned flap + bone re-contouring) + surface debridement using surgical gauzes soaked in saline + decontamination using 0.12 % CHX + 0.05 % cetylpyridinium chloride CPC	OHI/mechanical debridement + resective therapy (apical re-positioned flap + bone re-contouring) + surface debridement using surgical gauzes soaked in saline + decontamination using placebo solution	Test
		79 implants machined, rough- and medium-rough surfaces	Bone loss ≥2 mm				BOP: 80.4 (26.5) (BL) to 60.5 (30.1) % (12 months, implant level)
							PD: 6.6 (1.6) (BL) to 4.3 (2.1) mm (12 months, implant level)
							MBL: 4.3 (2.1) (BL) to 5.0 (2.5) mm (12 months, implant level)
							Control
							BOP: 79.7 (28.1) (BL) to 57.2 (29.0) % (12 months, implant level)
							PD: 5.5 (1.4) (BL) to 3.7 (0.8) mm (12 months, implant level)
							MBL: 3.6 (1.9) (BL) to 3.9 (2.0) mm (12 months, implant level)
							No sign. differences in BOP and PD reductions between groups
De Waal et al. [39]	RCT, parallel	44 patients	PD ≥5 mm, BOP + and/or suppuration	12 months	OHI/mechanical debridement + resective therapy (apical re-positioned flap + bone re-contouring) + surface debridement using surgical gauzes soaked in saline + decontamination using 0.12 % CHX + 0.05 % cetylpyridinium chloride	OHI/mechanical debridement + resective therapy (apical re-positioned flap + bone re-contouring) + surface debridement using surgical gauzes soaked in saline + decontamination using 2.0 % CHX	Test
		108 implants machined, rough- and medium-rough surfaces	Bone loss ≥2 mm				BOP: 82.1 (23.9) (BL) to 42.7 (34.2) % (12 months, implant level)
							PD: 4.7 (1.0) (BL) to 3.0 (0.7) mm (12 months, implant level)
							MBL: 4.0 (1.5) (BL) to 4.3 (1.7) mm (12 months, implant level)
							Control
							BOP: 74.2 (27.8) (BL) to 37.0 (35.5) % (12 months, implant level)
							PD: 5.0 (1.2) (BL) to 2.9 (0.7) mm (12 months, implant level)
							MBL: 4.1 (1.6) (BL) to 4.1 (1.7) mm (12 months, implant level)
							No sign. differences in BOP and PD reductions between groups
Papadopoulos et al. [41]	RCT, parallel	16 patients	PD ≥6 mm, BOP + and/or suppuration	6 months	Mechanical debridement + open flap surgery + mechanical debridement (plastic curettes) and cotton swabs soaked in saline	Mechanical debridement + open flap surgery + mechanical debridement (plastic curettes) and cotton swabs soaked in saline + 980 nm diode laser application	Test
		16 implants	Radiographic bone loss ≥2 mm				BOP: 81.2 (BL) to 23.8 % (6 months, implant level)
							PD: 5.92 (BL) to 4.44 mm (6 months, implant level)
							Control
							BOP: 81.2 (BL) to 23.8 % (6 months, implant level)
							PD: 5.52 (BL) to 4.31 mm (6 months, implant level)
							Sign. BOP and PD reductions in both groups at 6 months

*BL* baseline, *BOP* bleeding on probing, *CCT* non-randomized controlled clinical study, *CHX* chlorhexidine digluconate, *MBL* marginal bone level, *OHI* oral hygiene instructions, *PD* probing pocket depth, *RCT* randomized controlled clinical study, *SBI* sulcus bleeding index

^a^Subgroup analysis of *n* = 17 implants

^b^Subgroup analysis of *n* = 16 implants
- o Surgical treatment of peri-implantitis—adjunctive resective therapy (1 RCT) (Table 10)

**Table 10:** Included studies—surgical treatment of peri-implantitis: adjunctive resective therapy

Publication	Design	Population	Case definition	Period	Test	Control	Mean (SD) outcome
Romeo et al. [42, 57]	RCT, parallel	17 patients	Suppuration or sulcus bleeding, PD >4 mm horizontal peri-implant translucency	36 months systemic antibiotic medication (Amoxicillin for 8 days)	Full mouth disinfection/mechanical debridement + resective therapy (apical re-positioned flap + bone re-contouring) + decontamination using metronidazole + tetracycline hydrochloride (3 min) + implantoplasty using diamond and arkansas burs/silicone polishers	Full mouth disinfection/mechanical debridement + resective therapy (apical re-positioned flap + bone re-contouring) + decontamination using metronidazole + tetracycline hydrochloride (3 min)	Test
		22 implants rough surfaces					BOP: 2.83 (0.47) (BL) to 0.5 (0.69) (24 months^a^, implant level) PD: 5.79 (1.69) (BL) to 3.58 (1.06) mm (24 months^a^, implant level)
							MBL: 0.0–0.01 mm (36 months, implant level)
							Control
							BOP: 2.86 (0.35) (BL) to 2.33 (0.74) (24 months^a^, implant level)
							PD: 6.52 (1.62) (BL) to 5.5 (1.47) mm (24 months^a^, implant level)
							MBL: 1.44–1.54 mm (36 months, implant level)

*BL* baseline, *BOP* bleeding on probing, *MBL* marginal bone loss, *PD* probing pocket depth, *RCT* randomized controlled clinical study

^a^All patients of the control group were discontinued from the study due to persistent clinical signs of inflammation
- o Surgical treatment of peri-implantitis—adjunctive augmentative therapy (4 RCTs, 4 CCTs) (Table 11)

**Table 11:** Included studies—surgical treatment of peri-implantitis: adjunctive augmentative therapy

Publication	Design	Population	Case definition	Period	Test	Control	Mean (SD) outcome
Schwarz et al. [48, 51, 54]	RCT, parallel	20 patients	PD >6 mm, BOP + and/or pus	4 years nonsubmerged healing	OHI + initial nonsurgical therapy	OHI + initial nonsurgical therapy	Test
		21 implants machined and medium-rough surfaces	Intrabony defect >3 mm		Open flap surgery + mechanical debridement (plastic curettes) + nanocrystalline hydroxyapatite paste	Open flap surgery + mechanical debridement (plastic curettes) + bovine-derived xenograft + native collagen barrier membrane	BOP reduction: 32 % (4 years, subject level)
							PD reduction: 2.5 (0.9) mm (4 years, subject level)
							Control
							BOP reduction: 51 % (4 years, subject level)
							PD reduction: 1.1 (0.3) mm (4 years, subject level)
							BOP and PD reductions sign. higher at control sites
Deppe et al. [40]	CCT, parallel	32 patients	PD ≥5 mm, BOP + progressive vertical bone loss	5 years	Group 2 OHI + open flap^a^ surgery + air polishing + carbon dioxide laser (cw mode, 2.5 W, 12 × 5 s) decontamination + beta tricalcium phosphate + cortical bone chips harvested from the retromoar area (50:50) + nonresorbable synthetic barrier membrane	Group 4 OHI + open flap^b^ surgery + air polishing + beta tricalcium phosphate + cortical bone chips harvested from the retromoar area (50:50) + nonresorbable synthetic barrier membrane	Test
		73 implants machined, rough- and medium-rough surfaces					SBI: 0.5 (0.8) (BL) to 2.1 (1.4) (48 months, implant level)
							PD: 4.8 (1.4) (BL) to 2.5 (1.1) mm (48 months, implant level)
							Control
							SBI: 1.2 (0.6) (BL) to 1.9 (1.0) (48 months, implant level)
							PD: 5.7 (1.4) (BL) to 2.5 (1.4) mm (48 months, implant level)
							Comparable outcomes in both groups
Roos-Jansaker et al. [45–47]	CCT, parallel	25 patients	BOP + and/or pus	5 years transmucosal healing systemic antibiotic medication (amoxicillin + metronidazole for 10 days)	Removal of the suprastructure	Removal of the suprastructure	Test
		45 implants machined and medium-rough surfaces	Bone loss ≥3 threads one- to four-wall defects		Open flap surgery + debridement + decontamination using hydrogen peroxide 3 % algae derived xenograft + resorbable synthetic barrier membrane	Open flap surgery + debridement + decontamination using hydrogen peroxide 3 % algae derived xenograft +	PD reduction: 3.0 (2.4) mm (5 years, implant level)
							Radiographic defect fill: 0.1 (0.5) mm (5 years, implant level)
							Control
							PD reduction: 3.3 (2.0) mm (5 years, implant level)
							Radiographic defect fill: 0.1 (0.5) mm (5 years, implant level)
							Comparable defect fill and BOP reductions in both groups
Schwarz et al. [53]	CCT, parallel	27 patients	PD >6 mm, BOP + and/or pus	12 months nonsubmerged healing	Circumferential-type (Ie) defects OHI + initial nonsurgical therapy	Buccal dehiscence-type defects with a semicircular (Ib) or circular component (Ic)	Test Ib
		27 implants machined and medium-rough surfaces	Intrabony defect >3 mm		Open flap surgery + mechanical debridement (carbon curettes) + decontamination (cotton pellets soaked in saline)	OHI + initial nonsurgical therapy Open flap surgery + Mechanical debridement (carbon curettes) + decontamination (cotton pellets soaked in saline)	BOP reduction: 38.9 (16.6) % (12 months, subject level)
			Supracrestal component ≤1 mm		Bovine-derived xenograft + native collagen barrier membrane	Bovine-derived xenograft + native collagen barrier membrane	PD reduction: 1.6 (0.9) mm (12 months, subject level)
							Test Ic
							BOP reduction: 25.9 (14.7) % (12 months, subject level)
							PD reduction: 1.6 (0.7) mm (12 months, subject level)
							Control Ie
							BOP reduction: 61.1 (16.7) % (12 months, subject level)
							PD reduction: 2.7 (0.7) mm (12 months, subject level)
							Sign. difference in BOP reductions between Ic and Ie
Rocuzzo et al. [44]	CCT, parallel	26 patients	PD ≥6 mm	12 months nonsubmerged healing simultaneous connective tissue graft at sites lacking keratinized mucosa systemic antibiotic medication (amoxicillin + clavulanic acid for 6 days)	SLA surfaced implants	TPS surfaced implants	Test
		26 implants rough and medium-rough surfaces	Crater-like (intrabony) defects		OHI	OHI	BOP reduction: 60.4 % (12 months, subject level)
					Open flap surgery + mechanical debridement (plastic curettes) + decontamination (24 % EDTA and 1 % CHX gel) + bovine-derived xenograft	Open flap surgery + mechanical debridement (plastic curettes) + decontamination (24 % EDTA and 1 % CHX gel) + bovine-derived xenograft	PD reduction: 3.4 (1.7) mm (12 months, subject level)
							Radiographic defect fill: 1.9 (1.3) mm (12 months, subject level)
							Control
							BOP reduction: 33.9 % (12 months, subject level)
							PD reduction: 2.1 (1.2) mm (12 months, subject level)
							Radiographic defect fill: 1.6 (0.7) mm (12 months, subject level)
							BOP and PD reductions sign. higher in the test group
Schwarz et al. [49, 50, 52]	RCT, parallel	17 patients	PD >6 mm, BOP + and/or pus	4 years nonsubmerged healing	OHI + initial nonsurgical therapy	OHI + initial nonsurgical therapy	Test
		17 implants machined and medium-rough surfaces	Intrabony defect >3 mm		Open flap surgery + debridement + decontamination using an Er:YAG laser device (cone-shaped glass fibre tip) at 11.4 J/cm^2^ implantoplasty at bucally and supracrestally exposed implant parts	Open flap surgery + Mechanical debridement (plastic curettes) + decontamination (cotton pellets soaked in saline) implantoplasty at bucally and supracrestally exposed implant parts	BOP reduction: 71.6 (24.9) % (4 years, subject level)
			Supracrestal component >1 mm		Bovine-derived xenograft + native collagen barrier membrane at intrabony components	Bovine-derived xenograft + native collagen barrier membrane at intrabony components	PD reduction: 1.3 (1.8) mm (4 years, subject level)
							Control
							BOP reduction: 85.2 (16.4) % (4 years, subject level)
							PD reduction: 1.2 (1.9) mm (4 years, subject level)
							BOP and PD reductions comparable in both groups
Aghanzadeh et al. [43]	RCT, parallel	45 patients	PD ≥2 mm, BOP + and pus	12 months nonsubmerged healing systemic antibiotic medication (Azithromycin for 4 days)	Open flap surgery + mechanical debridement (titanium instruments) + decontamination using hydrogen peroxide 3 % cortical bone chips harvested from the mandibular ramus + resorbable synthetic barrier membrane	Open flap surgery + mechanical debridement (titanium instruments) + decontamination using hydrogen peroxide 3 % bovine-derived xenograft + resorbable synthetic barrier membrane	Test
		75 implants medium-rough surfaces	Bone loss ≥2 mm				BOP reduction: 44.8 (6.3) % (12 months, implant level)
			Angular defects ≥3 mm in depth				PD reduction: 2.0 (0.3) mm (12 months, implant level)
							Radiographic bone level gain: 0.2 (0.3) mm (12 months, implant level)
							Control
							BOP reduction: 50.4 (5.3 %) (12 months, implant level)
							PD reduction: 3.1 (0.2)mm (12 months, implant level)
							Radiographic bone level gain: 0.8 (0.4) mm (12 months, implant level)
							PD reductions and bone level gains were significantly higher at control sites
Wohlfahrt et al. [55, 58]	RCT, parallel	33 patients	PD ≥5 mm, BOP + intrabony defects ≥4 mm	12 months submerged healing for 6 months	Open flap surgery + mechanical debridement (titanium curettes) + conditioning using 24 % ethylenediaminetetraacetic acid gel (2 min) + augmentation of intrabony defect components using porous titanium granules	Open flap surgery + mechanical debridement (titanium currettes) + conditioning using 24 % ethylenediaminetetraacetic acid gel (2 min)	Test
		33 implants medium-rough surfaces					BOP reduction: 0.38 (2.1) % (12 months, implant level)
							PD reduction: 1.7 (1.7) mm (12 months, implant level)
							Radiographic defect fill: 57.0 (45.1) % (12 months, implant level)
							Control
							BOP reduction: 0.56 (2.9) % (12 months, implant level)
							PD reduction: 2.0 (2.3) mm (12 months, implant level)
							Radiographic defect fill: −14.8 (83.4) % (12 months, implant level) no sign. reductions in BOP scores in both groups comparable reductions in MMP-8 and bone level markers

*BL* baseline, *BOP* bleeding on probing, *CCT* non-randomized controlled clinical study, *CHX* chlorhexidine dugluconate, *MMP-8* matrixmetalloproteinase-8, *PD* probing pocket depth, *RCT* randomized controlled clinical study, *SBI* sulcus bleeding index, *SLA* sand blasted and acid etched, *TPS* titanium plasma flamed

^a^Subgroup analysis of *n* = 11 implants

^b^Subgroup analysis of *n* = 13 implants

#### Nonsurgical treatment of peri-implant mucositis

The case definitions markedly differed among the studies investigated. While three studies considered mucosal inflammation in the absence of radiographic bone loss [15–17], four studies also accepted a bone resorption of up to 3 mm for defining peri-implant mucositis [18–21]. Moreover, these studies used several clinical parameters to assess mucosal inflammation, employed various oral hygiene instructions (OHI) and defined different intervals for maintenance care. Despite significant improvements in all of the clinical parameters investigated, test (i.e. alternative or adjunctive methods for biofilm removal, adjunctive antiseptic therapy, or adjunctive antibiotic therapy) and control treatments were commonly associated with residual gingival index (GI), BI, and/or BOP scores at 3 to 12 months after therapy (Tables 3, 4 and 5).

##### Alternative or adjunctive measures for biofilm removal

Three studies reported on alternative or adjunctive measures for biofilm removal (Table 3).

In particular, one RCT and one CCT compared the clinical efficacy of adjunctive air polishing (glycine powder) to OHI and mechanical debridement using either ultrasonic scalers or hand instruments [15, 18]. Both test and control groups were associated with significant improvements in mean BI and PD scores after therapy. When evaluating absolute values at 6 months, mean BI and PD scores were significantly lower following adjunctive air polishing to teflon curettes [18]. One RCT compared a repeated (3 and 6 months) monotherapy using an air abrasive device to ultrasonic scaling. After a healing period of 12 months, both groups revealed comparable BOP reductions and frequencies of diseased sites [21] (Table 3).

##### Adjunctive antiseptic therapy

Three RCTs reported on the adjunctive antispectic therapy to OHI and mechanical debridement [17, 20, 22] (Table 4).

In particular, one RCT assessed the adjunctive application of phosphoric acid gel to carbon curettes and rubber cup polishing, which was provided once every month in both groups. At 5 months, test sites revealed a significantly higher reduction in mean gingival index (GI) (modified, but not specified) and colony-forming units when compared with control sites, respectively [22]. Porras et al*.* [20] compared OHI + mechanical debridement with and without local pocket irrigation using chlorhexidine digluconate (CHX) + topical CHX gel application + CHX mouthwash (twice for 10 days). At 3 months, mean mucosal bleeding (mBI), BOP scores and microbiological parameters did not significantly differ between test and control groups. However, the test group revealed a significantly higher change in mean PD scores [20]. In another RCT, topical CHX gel application + full mouth disinfection + CHX mouthrinse (2×/day) and tonsil spraying (1×/day) for 14 days was compared with OHI + mechanical debridement (plastic scaler + polyetheretherketone-coated ultrasonic instruments) + full mouth scaling alone. While both treatment procedures were associated with significant PD reductions at 8 months, the BOP scores did not significantly differ to baseline in both groups [17] (Table 4).

##### Adjunctive antibiotic therapy

Two studies reported on the adjunctive antibiotic (i.e. local or systemic) therapy to OHI and mechanical debridement [16, 19] (Table 5).

In particular, one RCT compared the adjunctive local delivery of tetracycline HCl (25 %) fibres for 10 days (test) to mechanical debridement alone (control). A complete or partial fibre loss was noted at three implants after 7 to 10 following application. While test sites were associated with marked BOP improvements, control sites revealed a further increase of BOP scores at 3 months [16]. In another RCT, a total of 45 patients were randomly allocated to either OHI + mechanical debridement (titanium curettes + rubber polishing) + systemic antibiotic medication (Azithromycin® 500 mg day 1 and 250 mg days 2–4) (test), or OHI + mechanical debridement alone (control). The subject-based, per-protocol analysis at 6 months did not reveal any significant differences between test and control groups for all clinical and microbiological parameters investigated [19] (Table 5).

#### Nonsurgical treatment of peri-implantitis

In all studies investigated, peri-implantitis was commonly defined by BOP and a radiographic bone loss. However, the reference points (i.e. baseline radiographs) and thresholds used to identify bone level changes were either not specified [23–25] or exhibited large variations [26–34]. Radiographic bone level changes as treatment outcome were merely assessed in three studies [28–30].

Despite significant improvements in all of the clinical and microbiological parameters investigated, test (i.e. alternative methods for biofilm removal, adjunctive antiseptic therapy, or adjunctive antibiotic therapy) and control treatments were commonly associated with residual BI and BOP scores at 3 to 12 months after therapy (Tables 6, 7 and 8).

##### Alternative measures for biofilm removal

Six RCTs (corresponding to 7 publications) reported on the efficacy of alternative measures for biofilm removal (Table 6). In particular, two studies employed the same type of an ultrasonic device used with a hydroxyapatite fluid polish [28, 31], while two studies reported on erbium-doped yttrium aluminum garnet (Er:YAG) laser monotherapy [24, 25] and two publications on glycine powder air polishing [27, 33]. One study compared Er:YAG laser monotherapy versus air polishing [30].

At 3 months after therapy, nonsurgical ultrasonic debridement was associated with a reduction in mean BOP scores, whereas these values further increased at control sites (i.e. carbon fibre curettes). However, these differences, as well as those noted for mean PD and radiographic bone level changes, did not reach statistical significance between groups [28]. Similarly, when comparing ultrasonic scaling with mechanical debridement using titanium curettes, Renvert et al*.* [31] also failed to identify any significant between group differences in mean BI and PD reductions at 6 months. Furthermore, both procedures did not reduce bacterial load [35] (Table 6).

In two RCTs, the efficacy of Er:YAG laser monotherapy was compared to that of mechanical debridement using carbon fibre curettes + adjunctive local antiseptic CHX irrigation/application [24, 25]. After 6 months of healing, Er:YAG laser application was associated with significantly lower mean BOP scores than the control treatment. However, these improvements failed to reach statistical significance at 12 months, particularly at advanced sites [24, 25] (Table 6). In one RCT, glycine powder air polishing resulted in a significantly higher reduction of mean BOP scores at 3, 6, and 12 months when compared with mechanical debridement + local antiseptic therapy using CHX. The application of this specific air abrasive device was not associated with any emphysema formation or complications in peri-implant wound healing [27, 33]. At more advanced sites, Er:YAG laser monotherapy and glycine powder air polishing resulted in comparable BOP/PD reductions and crestal bone level changes, but failed to reduce bacterial load [36] (Table 6).

##### Adjunctive antiseptic therapy

One multicenter RCT reported on the adjunctive antispective therapy to ultrasonic debridement (Table 7). In particular, a CHX containing matrix was repeatedly applied at 2, 4, 6, 8, 12 and 18 weeks until PD was reduced to ≤5 mm. At 6 months, CHX chips resulted in a significantly higher PD reduction than the placebo chips [23].

##### Adjunctive antibiotic therapy

Three RCTs reported on the adjunctive local antibiotic therapy to mechanical debridement (Table 8). In particular, minocycline microspheres were either applied once [32] at baseline or repeatedly [29] at 30 and 90 days and compared with local antispectic therapy using CHX gel (1.0 %). At 12 months, minocycline was associated with significantly higher BOP (refers to a repeated application) and PD (refers to a single application) reductions when compared with the control group. The radiographic (refers to a repeated application) and microbiological analyses failed to reveal any significant differences between both groups. Similar clinical outcomes were also noted when doxycycline hyclate was used as an adjunct to mechanical debridement [26].

In one RCT, adjunctive local antibiotic therapy (minocycline microspheres) was compared to adjunctive antimicrobial photodynamic therapy. At 12 months, both test and control groups were associated with significant but comparable clinical, microbiological and immunological improvements [34, 37] (Table 8).

The weighted mean (WM) BOP and PD reductions following conventional nonsurgical treatment (referring to the control groups in respective studies) [23–27] amounted to 31.12 % [SE = 9.14; 95 % CI (12.20, 49.05)] and 0.71 mm [SE = 0.32; 95 % CI (0.07, 1.35)], respectively. The weighted mean (WM) BOP and PD reductions for alternative/adjunctive measures (i.e. air polishing, aPDT, CHX chip, doxycycline, Er:YAG laser) [23–27] amounted to 42.85 % [SE = 9.24; 95 % CI (24.70, 60.97)] and 0.87 mm [SE = 0.29; 95 % CI (0.29, 1.44)], respectively.

#### Surgical treatment of peri-implantitis

Twelve studies (18 publications) reported on the surgical treatment of peri-implantitis, employing either alternative measures for surface decontamination (3 RCTs and 1 CCT) [38–41], adjunctive resective (1 RCT) [42] or augmentative (4 RCTs and 4 CCTs) [40, 43–55] therapy. In these studies, peri-implantitis was commonly defined by BOP and a radiographic bone level changes. However, the thresholds used to assess bone loss revealed large variations and defect configurations (i.e. supra-/intrabony defects) [56] were rarely reported (Tables 9, 10 and 11).

##### Alternative measures for surface decontamination

In one CCT, Deppe et al*.* [40] assessed the clinical efficacy of carbon dioxide laser decontamination used as an adjunct to resective flap surgery + air polishing (control). While the test treatment improved the clinical outcomes over the control measure at 4 months, mean SBI and PD values were comparable in both groups at about 5 years (Table 9).

In further two studies, De Waal et al*.* [38, 39] employed an open flap debridement using gauzes soaked in sterile saline + bone re-contouring + apical flap re-positioning and compared one test (0.12 % CHX + 0.05 % cetylpyridinium chloride) and two control (placebo solution or 2.0 % CHX) measures for surface decontamination. At 12 months, the test and both control procedures were associated with marked but comparable reductions in mean BOP and PD scores, respectively. Furthermore, between group comparisons failed to reveal any significant differences in mean marginal bone loss after therapy [38, 39]. Similarly, Papadopoulos et al*.* [41] also failed to reveal any significant clinical improvements in mean BOP and PD scores at 6 months, when a 980-nm diode laser was used as an adjunct to mechanical open flap debridement (Table 9).

##### Adjunctive resective therapy

One study assessed the clinical efficacy of an implantoplasty (diamond/arkansas burs + silicone polishers) when used as an adjunct to open flap debridement + bone re-contouring + apical flap re-positioning [42]. At 24 months, all patients from the control group had to be discontinued from the study due to persistent active signs of peri-implant inflammation. This was associated with elevated mBI and PD scores when compared with the test group. On the contrary, resective therapy resulted in significantly higher mean mucosal recessions (1.64 ± 1.29 vs. 2.3 ± 1.45 mm) but no pseudopocket formation [42], while test sites were associated with stable radiographic bone levels at 3 years, the interproximal bone loss at control sites amounted to 1.45–1.54 mm [57] (Table 10).

The calculated WM BOP [38, 39, 55] and PD [38–40, 55] reductions following surgical treatment (i.e. open flap with and without soft tissue resection) amounted to 34.81 % [SE = 8.95; 95 % CI (17.25, 52.37)] and 1.75 mm [SE = 0.34; 95 % CI (1.08, 2.42)].

##### Adjunctive augmentative therapy

The clinical efficacy of adjunctive augmentative therapy to open flap debridement (titanium curettes + conditioning using 24 % ethylenediaminetetraacetic acid gel + submerged healing for 6 months) was merely assessed in one study [55]. Notably, 12/16 control and 13/16 test sites revealed a premature exposure during the submerged healing phase of 6 months. At 12 months after therapy, the application of porous titanium granules to the intrabony defect components resulted in a significantly higher percentage of radiographic defect fill when compared with open flap surgery alone. Moreover, the test group was associated with an increase in implant stability quotient, whereas these values further decreased at control sites. However, both groups revealed comparable PD reductions and only minor improvements in mean BOP scores [55]. The immunological analysis did not reveal any significant between differences in the reduction of MMP-8 levels or bone level markers [58] (Table 11). Four RCTs and four CCTs compared different augmentation protocols employing various methods for surface decontamination, bone fillers (i.e. alloplastic, xenogenic, autogenous) and barrier membranes (synthetic, native collagen) over a period of up to 5 years [40, 43–55]. The majority of these studies considered PD and BOP reductions as primary outcomes but also reported on radiographic defect fill (Table 11).

WM BOP [43, 49, 51, 53, 55] and PD [40, 43–55] reductions following adjunctive augmentative therapy amounted to 50.73 % [SE = 3.5; 95 % CI (43.87, 57.59)] and 2.20 mm [SE = 0.22; 95 % CI (1.76, 2.64)], respectively. The outcomes of therapy was mainly influenced by the type of bone filler (i.e. a slowly resorbing bovine-derived mineral was superior to autogenous bone and an alloplastic material), defect characteristics (i.e. circumferential-type defects were superior to dehiscence-type defects) and implant surface characteristics (i.e. moderately rough surfaces were superior to rough surfaces) (Table 11).

#### Meta-analysis

Meta-analysis to estimate the weighted mean difference (WMD) between test and control treatments was conducted on RCTs reporting on similar assessments of either absolute BOP or PD scores.

##### Nonsurgical treatment of peri-implant mucositis—adjunctive antiseptics/antibiotics

**Fig. 2:**
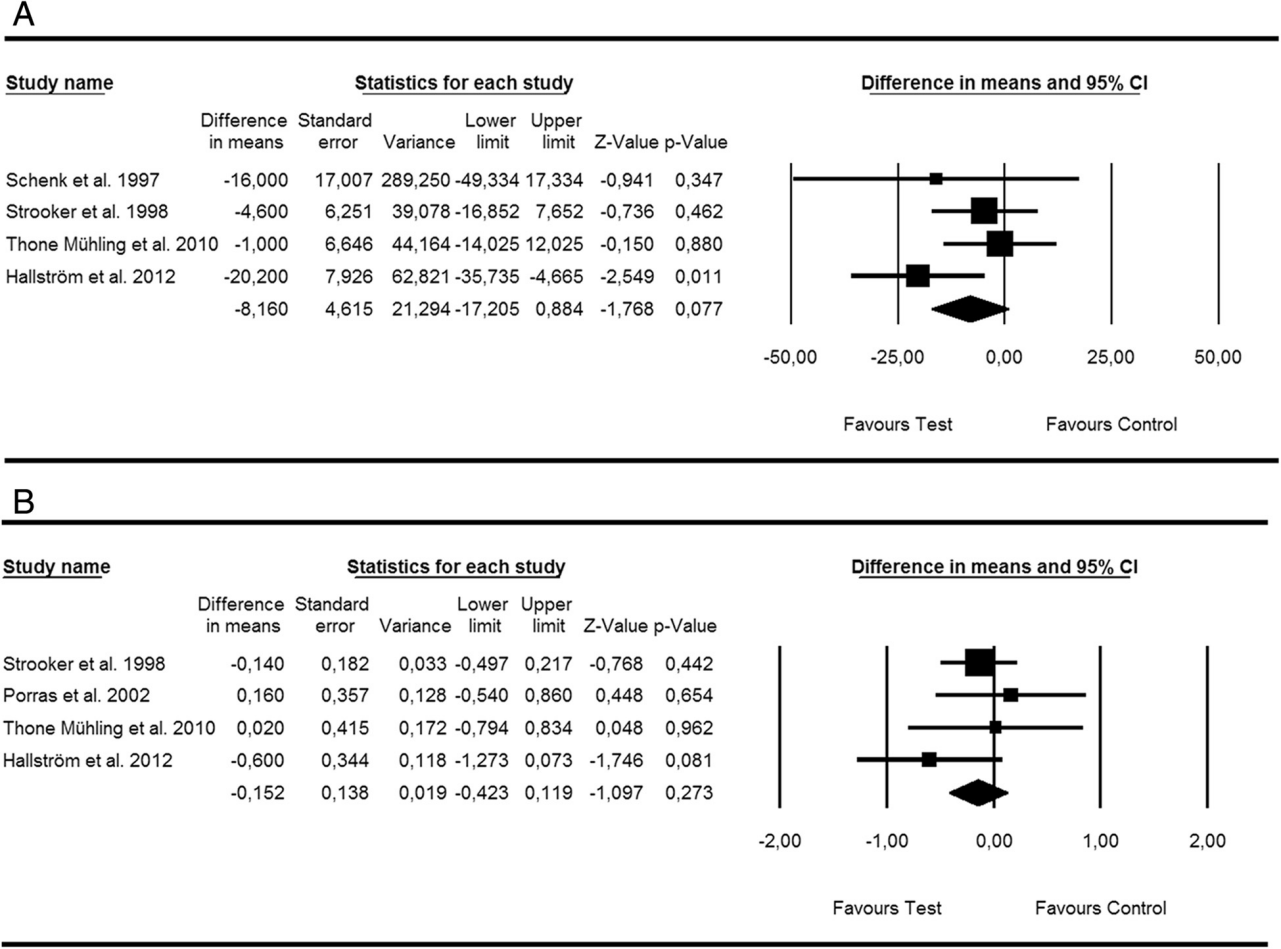
Forest plot indicating weighted mean difference (95 % CI) in the reduction of primary outcomes following nonsurgical treatment of peri-implant mucositis. **a** Adjunctive antiseptic/antibiotic therapy—BOP. **b** Adjunctive antiseptic/antibiotic therapy—PD

Based on four and four studies, WMD in BOP [16, 17, 19, 22] and PD [17, 19, 20, 22] scores amounted to −8.16 % [SE = 4.61; *p* > 0.05; 95 % CI (−17.20, 0.88)] and −0.15 mm [SE = 0.13; *p* > 0.05; 95 % CI (−0.42, 0.11)], not favouring local antiseptic or antibiotic (i.e. local and systemic) therapy as an adjunct to mechanical debridement (*p* value for heterogeneity: 0.42, *I*^2^ = 0.0 % = low heterogeneity; *p* value for heterogeneity: 0.45, *I*^2^ = 0.0 % = low heterogeneity, respectively) (Fig. 2a, b).

Egger’s linear regression method revealed symmetrical plots for changes in BOP (*p* = 0.51) and PD (*p* = 0.69) thus suggesting the absence of publication bias.

##### Nonsurgical treatment of peri-implantitis—alternative methods for biofilm removal

**Fig. 3:**
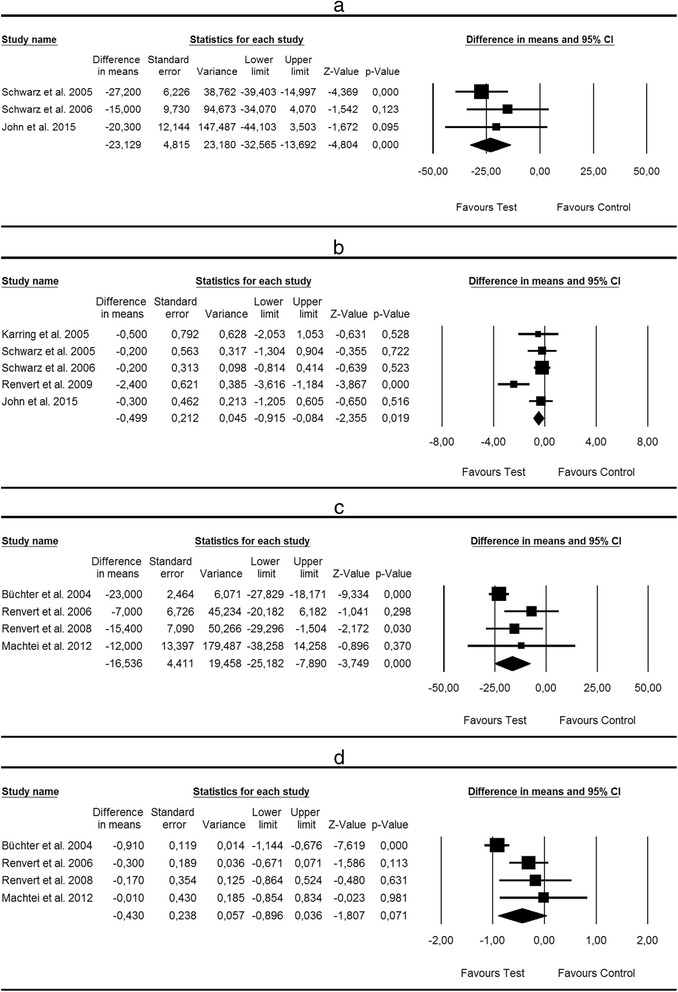
Forest plot indicating weighted mean difference (95 % CI) in the reduction of primary outcomes following nonsurgical treatment of peri-implantitis. **a** Alternative measures for biofilm removal—BOP. **b** Alternative measures for biofilm removal—PD. **c** Adjunctive antibiotic therapy—BOP. **d** Adjunctive antiseptic/antibiotic therapy—PD

Based on three studies [24, 25, 27], WMD in BOP scores between test and control groups amounted to −23.12 % [SE = 4.81; *p* < 0.001; 95 % CI (−32.56, −13.69)] favouring alternative methods (i.e. Er:YAG laser, glycine air polishing,) for biofilm removal over mechanical debridement (*p* value for heterogeneity: 0.55, *I*^2^ = 0.0 % = low heterogeneity; 0.39) (Fig. 3a). Based on five studies [24, 25, 27, 28, 31], WMD in PD scores between test and control groups amounted to −0.49 mm [SE = 0.21; *p* < 0.05; 95 % CI (−0.91, −0.08)] not favouring alternative methods (i.e. Er:YAG laser, glycine air polishing, ultrasonic system) for biofilm removal over mechanical debridement (*p* value for heterogeneity: 0.029, *I*^2^ = 62.8 % = substantial heterogeneity; 0.39) (Fig. 3b).

Egger’s linear regression method revealed symmetrical plots for changes in BOP (*p* = 0.41) and PD (*p* = 0.39) thus suggesting the absence of any publication bias.

##### Nonsurgical treatment of peri-implantitis—adjunctive antiseptic/antibiotic therapy

Based on four studies [23, 26, 29, 32], WMD in BOP scores between test and control groups amounted to −16.53 % [SE = 4.41; *p* < 0.001; 95 % CI (−25.18, −7.89)] favouring local antibiotic therapy as an adjunct to mechanical debridement (*p* value for heterogeneity: 0.113, *I*^2^ = 49.77 % = moderate heterogeneity) (Fig. 3c). WMD in PD scores between test and control groups amounted to −0.829 mm [SE = 0.51; *p* > 0.05; 95 % CI (−1.84, 0.18)] not favouring antiseptic/antibiotic therapy as an adjunct to mechanical debridement (*p* value for heterogeneity: 0.000, *I*^2^ = 87.37 % = considerable heterogeneity) (Fig. 3d).

Egger’s linear regression method revealed symmetrical plots for changes in BOP (*p* = 0.17) and PD (*p* = 0.07) thus suggesting the absence of any publication bias.

##### Surgical treatment of peri-implantitis - alternative measures for surface decontamination

**Fig. 4:**
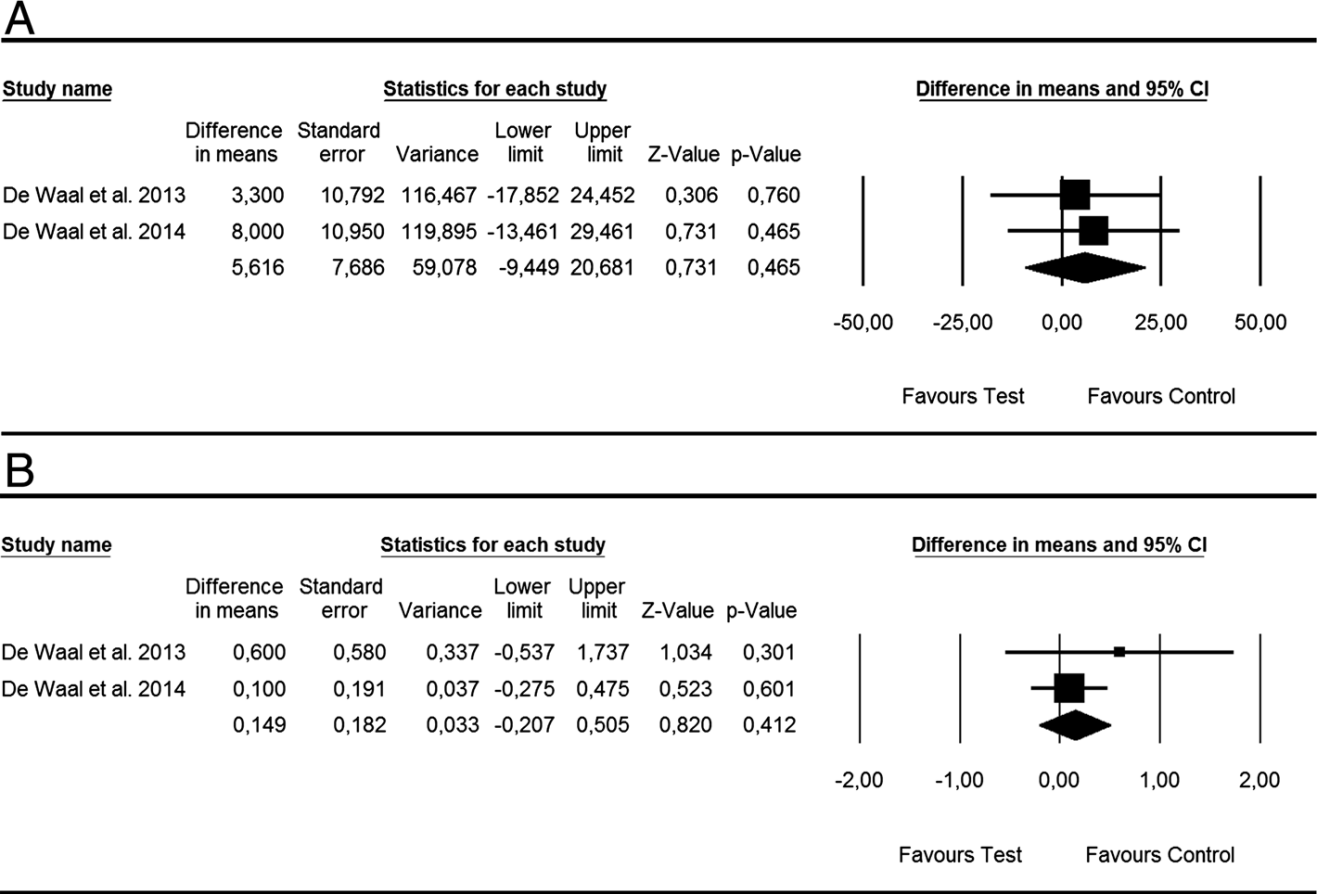
Forest plot indicating weighted mean difference (95 % CI) in the reduction of primary outcomes following surgical treatment of peri-implantitis. **a** Alternative measures for surface decontamination—BOP. **b** Alternative measures for surface decontamination—PD

Based on two studies [38, 39], WMD in BOP and PD scores between test and control groups amounted to 5.61 % [SE = 7.68; *p* > 0.05; 95 % CI (−9.44, 20.68)] and 0.22 mm [SE = 0.22; *p* > 0.05; 95 % CI (−0.20, 0.65)] not favouring alternative (i.e. CHX + CPC) over conventional (i.e. CHX) measures for surface decontamination (*p* value for heterogeneity: 0.76, *I*^2^ = 0.0 % = low heterogeneity; 0.60, *I*^2^ = 0.0 % = low heterogeneity, respectively) (Fig. 4a, b).

### Discussion

The present systematic review and meta-analysis was conducted to address the following focused question: “In patients with peri-implant mucositis and peri-implantitis, what is the efficacy of nonsurgical (i.e. referring to peri-implant mucositis and peri-implantitis) and surgical (i.e. referring to peri-implantitis) treatments with alternative or adjunctive measures on changing signs of inflammation compared with conventional nonsurgical and surgical treatments alone?”.

Basically, the literature search revealed that only a few studies considered appropriate test and control treatments needed to address the aforementioned focused question. In particular, this was true for 8 (7 RCTs and 1 CCT) [15–22] and 9 (9 RCTs) [23–26, 28, 29, 31–33] studies reporting on the nonsurgical treatment of peri-implant mucositis and peri-implantitis, as well as 5 RCTs [38, 39, 41, 42, 55] reporting on the surgical treatment of peri-implantitis. In addition, 5 RCTs and 4 CCTs not implementing appropriate control measures (i.e. mechanical/ultrasonic debridement or open flap debridement alone) but reporting on changes in primary outcomes were included for the estimation of the overall efficacy (referring to WM changes in BOP and PD scores) of nonsurgical [30, 34] and surgical [40, 43, 44, 46, 48, 52, 53] treatments of peri-implantitis. Moreover, it must be emphasized that the percentage across all included studies for high risk of bias items was 34.1 %, thus pointing to a need to improve the quality of reporting in future studies.

Within these limitations, the current data synthesis revealed that for the nonsurgical treatment of peri-implant mucositis, WMD in BOP [16, 17, 19, 22] and PD [15, 17, 19, 20, 22] scores amounted to −8.16 % and −0.15 mm, not favouring local antiseptic or antibiotic (i.e. local and systemic) therapy as an adjunct to mechanical debridement alone. Basically, these data corroborate the findings of a recent systematic review and meta-analysis, also indicating that adjunctive therapy may not improve the efficacy of professionally administered plaque removal in reducing BOP (i.e. local antiseptic or local/systemic antibiotics), GI and PD (i.e. local antiseptics, systemic antibiotics, air abrasive device) scores at mucositis sites [9]. When considering the present narrative data synthesis on the adjunctive [15] or alternative use of glycine powder air polishing [21], it was also noted that this device did not reveal any major improvements in BI/BOP scores or disease resolution over the respective control measures. In this context, it must be emphasized that BOP is the key parameter for the diagnosis of peri-implant mucositis [1], and the “resolution of peri-implant mucosal inflammation as evidenced by the absence of BOP” was the suggested endpoint following nonsurgical treatment of mucositis lesions [13]. All these data, taken together with the present findings support the view that OHI and mechanical debridement with or without polishing tools may be defined as a current standard of care for the management of peri-implant mucositis [6].

In contrast, for the nonsurgical treatment of peri-implantitis, WMD in BOP scores amounted to −16.53 % [23, 26, 29, 32] and 23.12 % [24, 25, 27], thus favouring either adjunctive local antibiotic therapy or alternative measures for plaque removal (i.e. Er:YAG laser or glycine powder air polishing) over respective control treatments. However, these improvements were not observed when analysing WMD in PD scores between test and control groups. Basically, these observations were also supported by the differences in the estimated WM BOP (31.12 vs. 42.85 %) and PD (0.71 vs. 0.87 mm) reductions noted following nonsurgical treatment of peri-implantitis using either conventional [23–27] or alternative/adjunctive measures (i.e. air polishing, aPDT, CHX chip, doxycycline, Er:YAG laser) [23–27], respectively.

Since the suggested endpoint following nonsurgical treatment of peri-implantitis is a “composite outcome of disease resolution including the absence of deep PD with bleeding and suppuration” [13], one has to critically emphasize the limited efficacy at “deep sites”. In particular, several studies reported on increasing BOP scores between 3 and 12 months following nonsurgical treatment of “severe” peri-implantitis sites using either mechanical debridement, adjunctive aPDT, Er:YAG laser monotherapy or glycine powder air polishing. The efficacy of all treatment procedures investigated was higher at “moderately” deep sites [24, 27, 33, 59]. In this context, however, one also has to realize that PD scores at implant sites may be influenced by a variety of different local factors, including the soft tissue thickness, vertical implant positioning, or a specific design of the implant-abutment connection (e.g*.* platform-switching). Accordingly, the classification of a “deep” pocket needs to be accomplished on an individual basis and disease severity should also consider “proportions of affected implants per patient in the presence of multiple implants” [6].

When further analysing the present results, it was also noted that nonsurgical treatment of peri-implantitis commonly failed to result in major microbiological improvements [32, 34–37], thus potentially explaining the frequency of residual BOP scores at respective sites.

At the time being, there is a lack of clinical studies aimed at comparing the efficacy of nonsurgical and surgical treatments of peri-implantitis. However, a preclinical study employing the ligature model has indicated that open flap debridement was associated with significant histological improvements in osseous defect fill and establishment of a new bone-to-implant when compared with nonsurgical treatments. The latter outcome was mainly influenced by the method of surface decontamination [60]. Accordingly, a “proven method of decontaminating the implant surface” has been defined as a critical component in surgical therapy [13]. However, the present qualitative and quantitative analysis has indicated that the clinical, radiographical and microbiological outcomes following either open flap debridement or surgical augmentative therapy were not influenced by the decontamination protocol, including chemical or photothermal (i.e. carbon dioxide, diode- or Er:YAG laser radiation) approaches [38–41, 49, 50, 52]. Moreover, two RCTs [38, 39] reported on an additional bone loss at 6 and 12 months after open flap debridement, thus indicating that disease resolution (i.e. “absence of deep probing pocket depths with bleeding and suppuration and no additional bone loss”) [13] was commonly not achieved.

The present data synthesis also revealed a lack of RCTs/CCTs implementing appropriate test and control groups to assess the efficacy of adjunctive resective or augmentative measures over open flap debridement alone. The available studies have indicated that resective surgery (i.e. apical re-positioned flap + bone contouring) + implantoplasty was more effective in obtaining and maintaining disease resolution over resective surgery alone [42, 57]. In contrast, surgical augmentative therapy of the intrabony defect component using porous titanium granules was associated with a significantly higher radiographic defect fill, but failed to improve a reduction in mean BOP and PD scores over the control treatment [55]. When considering the estimated WM BOP (50.73 vs. 34.81 %) [38, 39, 43, 49, 51, 53, 55] and PD (2.20 vs. 1.75 mm) [38–40, 43–55] reductions, the clinical outcomes obtained following adjunctive augmentative therapy tended to be improved when compared with surgical measures alone. However, it has to be realized that for the data synthesis in the latter group, surgical procedures with and without soft tissue resection were combined, and therefore, the interpretation of the overall performance of surgical measures without augmentative measures on PD reductions is difficult. Moreover, obvious variations in the surgical procedures, including different decontamination protocols, administration of prophylactic systemic antibiotics, and modes of healing (i.e. open vs. submerged) may not allow for a direct comparison of these estimated outcomes. Basically, the estimated WM BOP and PD reductions corroborate those calculations reported in a recent systematic review on reconstructive procedures for the management of peri-implantitis. When also case series were included in the meta-analysis, these values amounted to 45.8 and 2.97 mm [61]. Furthermore, the present qualitative analysis of the available data on surgical augmentative therapy have indicated, that the outcomes of therapy may be influenced by several local factors, mainly including the physicochemical properties of the bone filler [43, 48, 51, 54], the defect configuration [53], as well as implant surface characteristics [44]. Any beneficial effect of a resorbable synthetic barrier membrane could not be identified [45–47]. Nevertheless, the available evidence did not allow for any conclusive statements on the potential superiority of any particular augmentation protocol.

Finally, it must be emphasized that laser therapy, the application of bone grafts and barrier membranes were reported to be associated with the highest cost-effectiveness ratio (i.e. costs and proportions of lost implants) among 11 treatment procedures investigated [62].

## Conclusions

While OHI + mechanical debridement alone was found to be effective for the management of peri-implant mucositis, alternative/adjunctive measures may improve the efficacy over/of conventional treatments at peri-implantitis sites. Adjunctive resective and/or augmentative measures are promising; however, their beneficial effect on the clinical outcome of surgical treatments needs to be further investigated.

## Abbreviations

aPDT: antimicrobial photodynamic therapy
BOP: bleeding on probing
CAL: clinical attachment level
CCD: controlled clinical study
CHX: chlorhexidine digluconate
Er:YAG laser: erbium-doped yttrium aluminum garnet laser
GI: gingival index
mBI: (modified) bleeding index
MMP: matrixmetalloproteinase
MR: mucosal recession
OHI: oral hygiene instructions
PD: probing pocket depth
RCT: randomized controlled clinical study
WM: weighted mean changes
WMD: weighted mean differences
